# Polycyclic Aromatic Hydrocarbons Reciprocally Regulate IL-22 and IL-17 Cytokines in Peripheral Blood Mononuclear Cells from Both Healthy and Asthmatic Subjects

**DOI:** 10.1371/journal.pone.0122372

**Published:** 2015-04-10

**Authors:** Coline Plé, Ying Fan, Saliha Ait Yahia, Han Vorng, Laetitia Everaere, Cécile Chenivesse, Joanne Balsamelli, Imane Azzaoui, Patricia de Nadai, Benoit Wallaert, Gwendal Lazennec, Anne Tsicopoulos

**Affiliations:** 1 Institut National de la Santé et de la Recherche Médicale, U1019, F-59019, Lille, France; 2 Institut Pasteur de Lille, Center for Infection and Immunity of Lille, F-59019, Lille, France; 3 CNRS UMR 8204, F-59000, Lille, France; 4 Univ Lille Nord de France, F-59000, Lille, France; 5 Clinique des Maladies Respiratoires et Centre Hospitalier Régional et Universitaire de Lille, F-59037, Lille, France; 6 CNRS SysDiag—UMR3145 Cap delta, 1682 rue de la Valsière, F-34184, Montpellier Cedex 4, France; University Medical Center Freiburg, GERMANY

## Abstract

Pollution, including polycyclic aromatic hydrocarbons (PAH), may contribute to increased prevalence of asthma. PAH can bind to the Aryl hydrocarbon Receptor (AhR), a transcription factor involved in Th17/Th22 type polarization. These cells produce IL17A and IL-22, which allow neutrophil recruitment, airway smooth muscle proliferation and tissue repair and remodeling. Increased IL-17 and IL-22 productions have been associated with asthma. We hypothesized that PAH might affect, through their effects on AhR, IL-17 and IL-22 production in allergic asthmatics. Activated peripheral blood mononuclear cells (PBMCs) from 16 nonallergic nonasthmatic (NA) and 16 intermittent allergic asthmatic (AA) subjects were incubated with PAH, and IL-17 and IL-22 productions were assessed. At baseline, activated PBMCs from AA exhibited an increased IL-17/IL-22 profile compared with NA subjects. Diesel exhaust particle (DEP)-PAH and Benzo[a]Pyrene (B[a]P) stimulation further increased IL-22 but decreased IL-17A production in both groups. The PAH-induced IL-22 levels in asthmatic patients were significantly higher than in healthy subjects. Among PBMCs, PAH-induced IL-22 expression originated principally from single IL-22- but not from IL-17- expressing CD4 T cells. The Th17 transcription factors *RORA* and *RORC* were down regulated, whereas AhR target gene *CYP1A1* was upregulated. IL-22 induction by DEP-PAH was mainly dependent upon AhR whereas IL-22 induction by B[a]P was dependent upon activation of PI3K and JNK. Altogether, these data suggest that DEP-PAH and B[a]P may contribute to increased IL22 production in both healthy and asthmatic subjects through mechanisms involving both AhR -dependent and -independent pathways.

## Introduction

Allergic asthma has strongly increased in the last decades in western countries and is considered mainly as a Th2 mediated disease. There is increasing evidence that environmental pollution contributes to this augmented prevalence [1]. In particular, we and others have shown that Polycyclic Aromatic Hydrocarbons derived from diesel exhaust particles (DEP-PAH) play an inflammatory and adjuvant role in the development and exacerbation of allergic inflammation by skewing the immune response towards a Th2 profile [2–5].

It is now clear that the biological response to many environmental pollutants is a direct consequence of their interactions with the Aryl hydrocarbon Receptor (AhR), a cytosolic transcription factor which binds exogenous ligands such as PAH or 2,3,7,8-tetrachlorodibenzo-p-dioxin (TCDD), as well as endogenous ligands derived from tryptophane metabolism such as 6-formylindolo[3,2-b] carbazole (FICZ). Recent data suggest that AhR may be a major transcription factor involved in the development of the Th17 and Th22 subsets [6–8]. The human Th17 subpopulation is characterized by the production of IL-17A, IL-17F, IL-22 and CCL20 [9], and the expression of RORα and RORC [10], whereas the Th22 subpopulation produces IL-22 but not IL-17A [7]. IL-17A and IL-22 are both involved in neutrophilic influx, airway smooth muscle cell proliferation and migration [11–14], and IL-22 is also involved in tissue repair and remodeling mechanisms [15]. In agreement with the presence of these endpoints in asthma, increased levels of IL-17A, IL-17F and IL-22 are found at the circulating and lung levels in allergic asthmatic patients as compared with controls [16–18].

Although AhR has been consistently involved in IL-22 production by T cells [7,19], controversial results have been obtained for IL-17A production. Indeed, some AhR ligands such as TCDD and FICZ have been shown to induce IL-17A production in mice [6–8] and to inhibit it in humans [7,19,20]. Moreover, AhR has been shown to favour IL-10-producing regulatory T cells according to other studies in both mice and humans [21,22]. These apparently contradictory results may relate to the ligand used (endogenous favouring IL17 versus exogenous favouring IL-10) [6,8], to the species [7,8], to the culture conditions (i.e. added cytokines) [19,23,24] but also to the cells expressing AhR. Indeed, besides CD4 T cells, CD8 T cells and dendritic cells also express AhR. Despite these discrepancies, these studies suggest a potential link between environmental pollutants able to bind to AhR, and the Th17/Th22 program. Therefore, we hypothesized that DEP-PAH, and some purified PAH previously shown to be involved in asthma, might affect, through their effects on AhR, IL-17 and IL-22 cytokine profiles.

In the present study, activated peripheral blood mononuclear cells from allergic asthmatics exhibited an increased Th17/Th22 type profile compared with nonallergic nonasthmatic subjects. Only some of the PAH tested including DEP-PAH- and Benzo[a]pyrene- (B[a]P) exacerbated IL-22 production by stimulated mononuclear cells, whereas IL-17 production was inversely down regulated in asthmatic as well as healthy subjects. The mechanisms of regulation of IL-22 induction were dependent upon AhR, and also involved Mitogen-activated protein kinase (MAPK) and Phosphotidylinositol 3-kinase (PI3K) pathways.

## Materials and Methods

### Human donors

Venous blood was collected from 16 healthy nonallergic nonasthmatic (NA) subjects, with negative skin prick tests and no specific IgE to common environmental allergens, and from 16 allergic asthmatic (AA) patients. All these patients had a clinician’s diagnosis of asthma, and exhibited positive skin prick tests and specific IgE (more than 3 KU/L) towards at least one allergen. Skin prick testing included house dust mite, grass and tree pollens, cat and dog danders as well as fungi. Exclusion criteria included upper or lower respiratory tract infection within one month of study, use of anti-inflammatory controller medications within four weeks of study, significant non-asthma pulmonary disease or other medical problems. All subjects were non-smokers. To exclude the potential confounding effects of steroid treatment, only intermittent asthmatic subjects taking only β2 agonists as needed, according to GINA guidelines, were included. Among potential confounding factors for IL-17 and IL-22 expression, we anticipated severity of the disease, recent infections and smoking, however we cannot exclude uncontrolled factors such as encountered environmental pollution or diet. The subject’s characteristics are shown in S1 Table. The study was approved by the Centre Hospitalier Régional Universitaire of Lille Ethical Review Committee (Number 2008/010) and all donors signed an informed consent form.

### Cell purification and culture

As there are some controversies about the effect of AhR ligands on the production of IL-17 which may relate to the use of particular single cell types (memory, naïve CD4 T cells, T cells during cytokine induced differentiation, etc.), we evaluated the effects of PAH in an environment where all these cell types are represented and can interact, in particular with dendritic cells, i.e. peripheral blood mononuclear cells (PBMCs). These cells were prepared from blood collected on heparin as previously described [25]. To activate Th17/Th22 cells, we used a surrogate of antigen stimulation, through CD3 combined with co-stimulation by CD28. Preliminary experiments were performed to determine the minimal dose of CD3 antibody allowing IL-17 production. Concentrations between 10μg/mL and 1ng/mL were tested which showed that the 100ng/mL concentration reproducibly induced production of IL-17 by PBMCs. Moreover preliminary kinetics experiments performed between 24 and 96hr after stimulation showed that the highest level of IL-17 was observed 72 hrs after stimulation, this time point was therefore chosen for the study. PBMCs (2.10^6^/ml) were stimulated in 48-well flat-bottomed microculture plates coated with the minimal anti-CD3 concentration (100ng/ml, OKT3 clone) and soluble anti-CD28 (2μg/ml; BD Biosciences) in complete Iscove's modified Dulbecco's medium (Invitrogen Corporation, Carlsbad, CA, USA), a medium previously shown to favour IL-17 production [26]. A mix of DEP-PAH standard reference material (SRM 1975, Interchim, Montluçon, France) as well as different purified PAH including the prototypical PAH Benzo[a]Pyrene (B[a]P), and two PAH previously shown to increase allergen-induced asthmatic responses [27,28], 9–10 Phenanthrene quinone (PHEQ), and anthracene (ANT) (Sigma-Aldrich, St Louis, MO, USA), were dissolved in 0.02% dimethyl sulfoxide (DMSO) and used at optimal non-cytotoxic doses (150ng/ml for SRM, 250ng/ml for B[a]P, 75nM for PHEQ and 1μM for ANT). In all experiments the cell viability was more than 95%, as assessed by trypan blue exclusion. DMSO was used as control. The supernatants and cells were collected after 72hr of culture. In some experiments, inhibitors as well as Th17 conditions were used. AhR antagonist CH-223191 (3μM; Calbiochem, Darmstadt, Germany), inhibitors of p38 kinase (SB203580, 1μM; Merck KGaA, Darmstadt, Germany), c-jun N-terminal kinase (JNK) (SP600125, 20 μM; Merck KGaA), MAP kinase kinase (MEK/ERK) (PD98059, 25 μM; Merck KGaA) or Phosphatidylinositol 3-kinase (PI3K) (LY294002, 10 μM: Merck KGaA) all used at non-cytotoxic concentrations were added to the cultures at the same time as the different stimuli. We used the AhR antagonist CH-223191 to inhibit AhR since it has been shown to exhibit a very high antagonist activity, while being not toxic [29]. For Th17 conditions stimulated PBMCs were cultured with IL-2, IL-6 (PeproTech, London, United Kingdom), IL-1β (eBiosciences, San Diego, CA, USA) and IL-23 (Miltenyi Biotec, Paris, France) all at 10ng/mL for 6 days.

### Cytokine secretion assays

Cytokines were measured by ELISA according to the manufacturer’s recommendations. IL-17A, IL-22, CCL20, IL-13, IL-5, IL-10, TGF-β1, periostin and CCL18 kits were from R&D systems (Minneapolis, MN, USA), IL-17F from eBiosciences and IFN-γ from BD Biosciences (Franklin Lakes, NJ, USA). The lower detection limit was 15.6 pg/ml for IL-17A, IL-17F and CCL20, 31.2 pg/ml for IL-22, IL-5, IFN-γ, and TGF-β, 93.8 pg/ml for IL-13, 375 pg/ml for periostin and 7.8 pg/ml for CCL18.

### Quantitative Real Time PCR

Quantitative real Time PCR was performed as previously described [30]. Briefly Total RNA was isolated using TRIzol reagent (Invitrogen) according to the manufacturer’s instructions. Reverse transcription was performed using random primers and M-MLV enzyme (Invitrogen). Real-time PCR quantification was performed using a SYBR Green approach (Light Cycler; Roche, Penzberg, Germany), and normalized to rs9 house keeping gene. The sequences of the oligonucleotides are shown in S2 Table.

### Flow Cytometry

IL-17 and IL-22 cell origin was determined by flow cytometry. Stimulated PBMCs (2.10^6^/ml) were incubated with HAP or DMSO with or without Th17 cytokines. At day 3 IL-2 was added again. At day 6, cells were stimulated by PMA (50ng/ml; Sigma-Aldrich) and Ionomycin (1μg/ml; Calbiochem) in the presence of Brefeldin A (10μg/ml; Sigma-Aldrich) for 4h. Cell surface and intra cellular staining was performed as previously described [31]. Specific cell populations were identified using the following combination of antibodies CD4-FITC^+^ (clone RPA-T4), CD8-PE^+^ (clone HIT8a) for T cells; CD3-FITC^**-**^ (clone HIT3a) CD56-PE^+^ (clone B159) for NK cells; iNKT-PE^+^ (clone 6B11) for iNKT cells; Lin2-FITC^**-**^ (clones NCAM 16.2, MΦP9, SK7, SJ25C1, L27) CD123-PE^+/**-**^ (clone 7G3) HLA-DR-V450^+^ (clone G46.6) for dendritic cells (DCs) (BD Biosciences) and intracellular staining using IL-17A-APC (clone eBio64DEC17), IL-22-PERCP eFluor710 (clone 22URTI) (eBiosciences). Mouse IgG1-APC, IgG1-PERCP eFluor710 isotypes (clone P3.6.2.8.1) were from eBiosciences. Living cells were gated using forward and side scatter properties, and further using livedead fixable aqua dead cell stain kit (Life technologies, St Aubin, France) and then cell populations were gated using the specific markers indicated above. Data were acquired on a BD LSR Fortessa flow cytometer using the DIVA software (version 6.1.3). Compensations were calculated using BD Compbeads with the automatic compensation programme. Data were expressed as percentage of cytokine positive cells among the considered population after subtraction of the control isotype.

### Statistical Analyses

Data were normally distributed (Shapiro wilk test) except for CCL18. For CCL18, data were analysed using non-parametric Friedman analysis of variance followed by Dunn’s post hoc test. For the other parameters, within NA/AA comparisons were performed using one way ANOVA followed by Holm-Sidak’s multiple comparison test, and between NA/AA comparisons using unpaired t-test. Statistical analyses were performed using GraphPad Prism 6 software (La Jolla, CA, USA). Values of p<0.05 were regarded as statistically significant. As this was an exploratory study, no adjustment for multiple testing was performed.

## Results

### Stimulated PBMCs from AA patients exhibit an exacerbated Th17 type cytokine and mRNA profile compared with NA subjects

**Fig 1:**
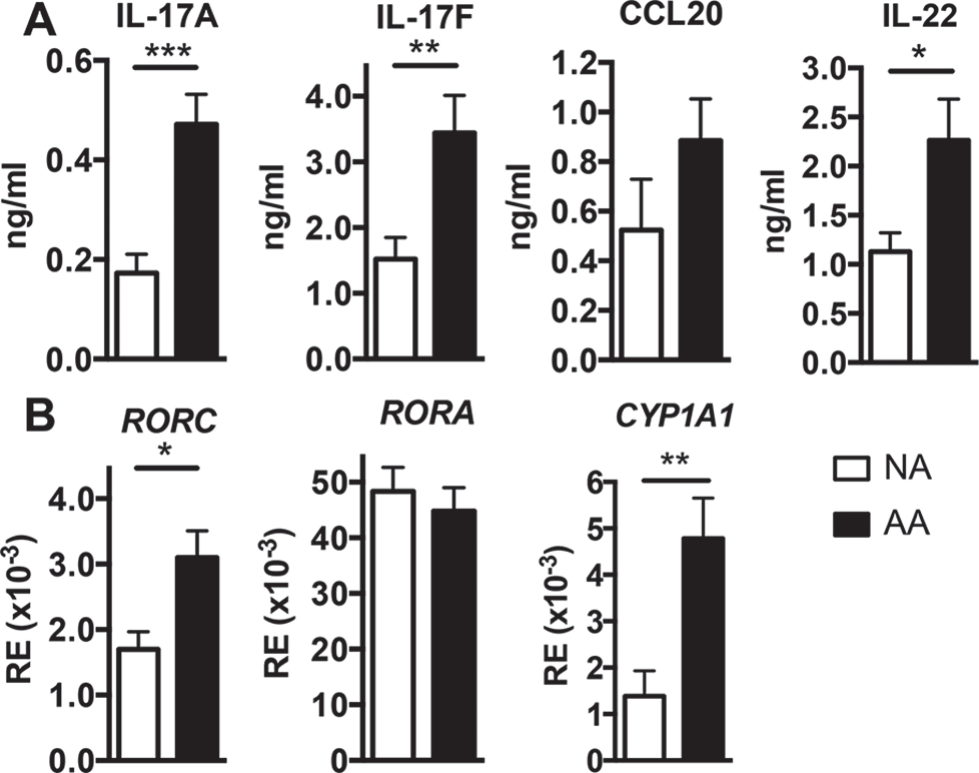
Th17/Th22 type profile of anti-CD3/CD28-activated peripheral blood mononuclear cells. **A** Cytokine secretion by activated peripheral blood mononuclear cells from nonallergic (NA) subjects (n = 10) and allergic asthmatic (AA) patients (n = 12), was evaluated at 72hr by ELISA and expressed as mean ± SEM ng/ml. **B** Gene mRNA level in activated peripheral blood mononuclear cells from NA subjects (n = 8) and AA patients (n = 11), was evaluated at 72hr by Q-RT-PCR and expressed as mean relative expression (RE) of 2^(-ΔCt) ± SEM, where the ΔCt value of the sample was determined by subtracting the Ct value of the target gene from the Ct value of the rs9 house keeping gene. *P<.05, **P<.01, and ***P<.001.

Previous studies have evidenced that allergic asthmatics exhibit higher levels of IL-17/IL-22 in the lung and sera than healthy subjects [16,17]. To evaluate if this difference was also found in PBMCs, Th17 type cytokine production was measured in anti-CD3/CD28 stimulated PBMCs. Indeed, almost no production of cytokines was found in the supernatants without anti CD3/CD28 stimulation, whatever the allergic status. IL-17A, IL-17F, and IL-22 but not CCL20, were increased in stimulated PBMCs from AA patients compared with NA subjects (Fig 1A). Levels of Th2- (IL-13, IL-5, periostin), Th1- (IFN-γ) and Tregulatory- (TGF-β1 and IL-10) type cytokines were not significantly different between NA and AA donors after anti CD3/CD28 stimulation (data not shown). Furthermore, the lineage-specifying Th17/Th22 transcription factors *RORC*, AhR target gene *CYP1A1*, but not *RORA* were upregulated in AA compared with NA subjects (Fig 1B).

### DEP-PAH and B[a]P drive reciprocal regulation of IL-17A and IL-22 production by PBMCs

**Fig 2:**
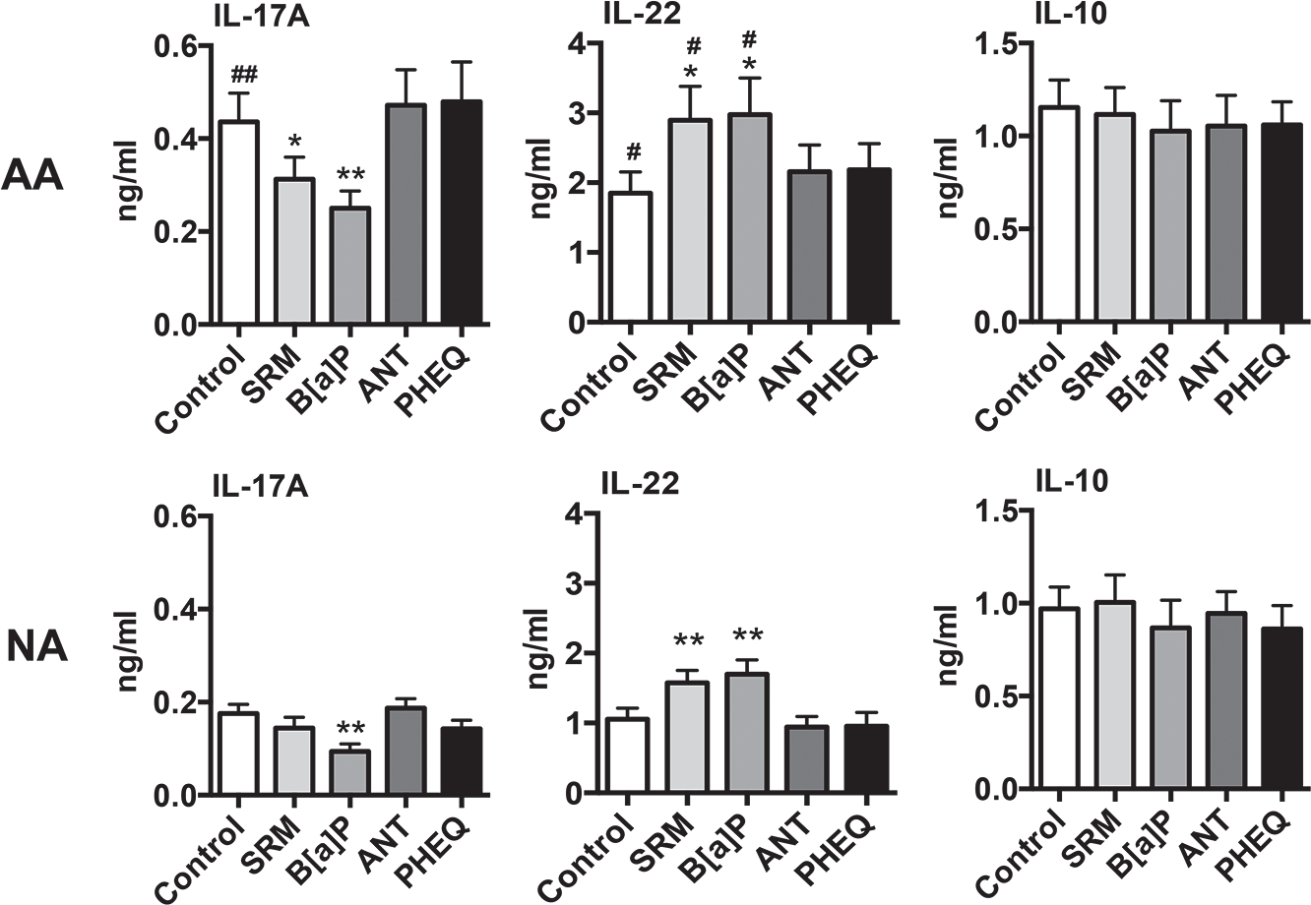
Cytokine profile of PAH-stimulated peripheral blood mononuclear cells in allergic asthmatics (AA) and nonallergic subjects (NA). IL-17A, IL-22 and IL-10 secretion by activated peripheral blood mononuclear cells from NA subjects (n = 10) and AA patients (n = 12) stimulated or not with different PAH for 72hr was determined by ELISA. Results are expressed as mean ± SEM. *P<.05 and **P<.01 versus control. ^#^ *P*<.05 and ^##^ *P*<.01 AA versus NA subjects.

**Fig 3:**
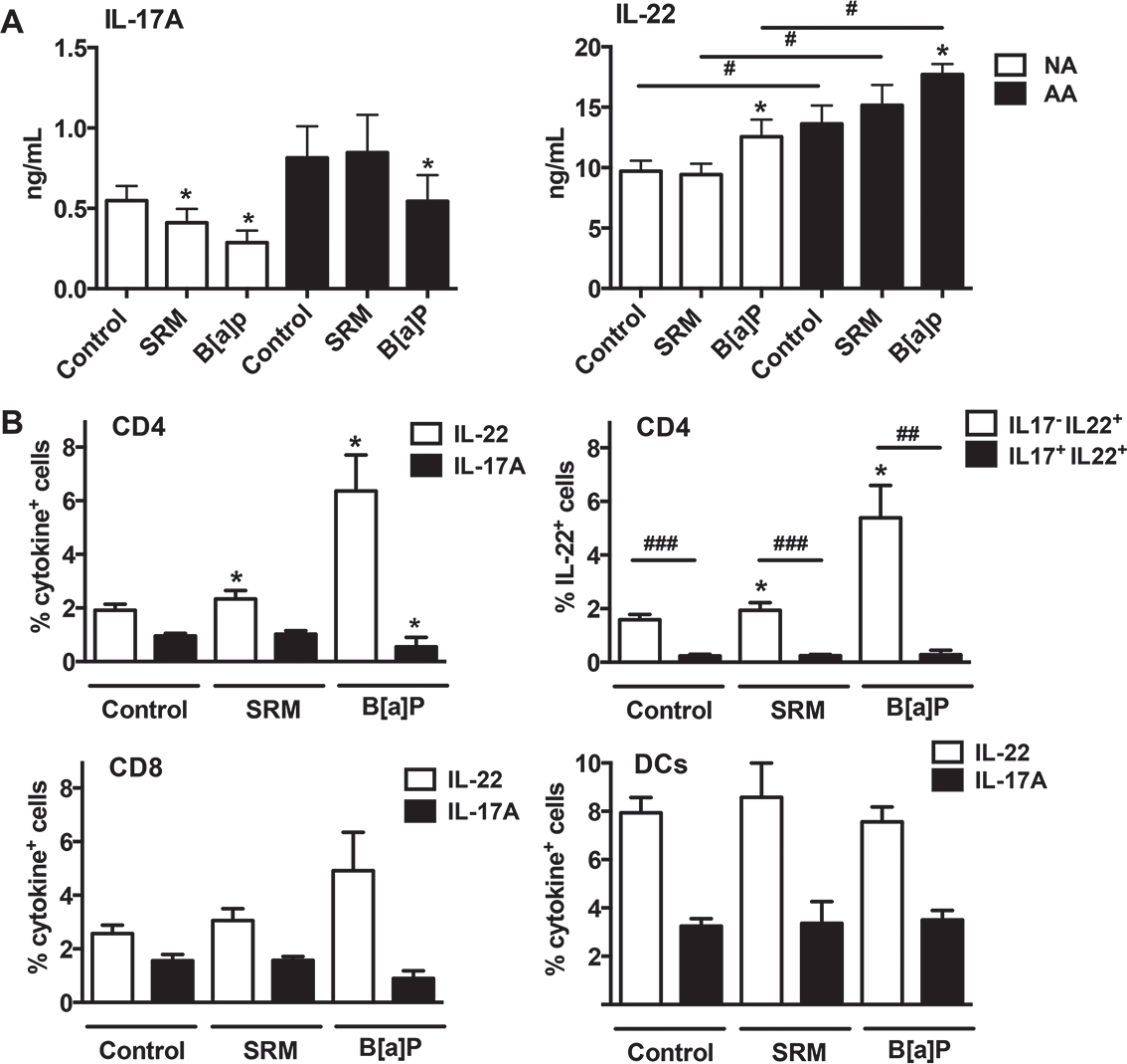
Cytokine profile and cell expression of PAH-stimulated peripheral blood mononuclear cells cultured in Th17 conditions. **A** IL-17A and IL-22 production by activated peripheral blood mononuclear cells from 6 nonallergic (NA) subjects and 6 allergic asthmatic (AA) patients stimulated or not with SRM or B[a]P for 6 days was determined by ELISA. Results are expressed as mean ± SEM. *P<.05 versus corresponding control. ^#^ *P*<.05 AA versus NA subjects. **B** Activated peripheral blood mononuclear cells were stimulated or not with SRM and B[a]P, stained with antibodies against cytokine and cell surface markers, and evaluated by flow cytometry. Results are expressed as mean ± SEM percentage of cytokine positive cell populations for n = 7–11 subjects. *P<.05 versus corresponding control, ^##^ *P*<.01, ^###^ *P*<.001.

To assess the ability of different PAH to modulate the production of Th17/Th22 type cytokines, activated PBMCs were co-incubated with SRM or with purified PAH. It is of note that PAH were not able to induce IL-17 or IL-22 production by unstimulated PBMCs. B[a]P decreased IL-17A secretion by stimulated PBMCs in both groups, whereas SRM decreased it only in AA patients (Fig 2). No effects were observed for ANT and PHEQ (Fig 2). IL-17F was slightly decreased in the presence of B[a]P, and only in AA patients (S1 Fig), and CCL20 was not modified (data not shown). An opposite effect was observed on IL-22 for SRM and B[a]P with increased secretion of IL-22 with a similar magnitude in healthy and asthmatic subjects, but with a final level significantly higher in AA patients than in NA subjects (Fig 2). PHEQ and ANT did not modify IL-22 production. As previously described [2–4], IL-13 production was significantly increased in DEP-PAH- and B[a]P- stimulated PBMCs from asthmatic (respectively 1.77 ± 0.27 and 1.73 ± 0.25 versus 1.28 ± 0.27 ng/ml for controls, p<0.05) but not from healthy subjects. Inhibition of IL-17A by PAH was not related to the induction of the regulatory cytokines IL-10 (Fig 2) or TGF-β1 (S1 Fig). In contrast, CCL18, a chemokine with immunoregulatory properties [25,31,32] was inhibited in response to B[a]P in both groups and by DEP-PAH in asthmatics (S1 Fig). PHEQ and ANT did not modify the production of IL-10 and CCL18 and therefore were not furthermore evaluated. To evaluate if the reciprocal regulation of IL17 and IL22 induced by PAH changed with the culture conditions, PBMCs were cultured in Th17 conditions. A similar pattern of IL-22 induction and IL-17 reduction by B[a]P was still observed, although with a higher level of cytokine production, and with significant differences between NA and AA subjects for IL-22 (Fig 3A). Collectively, these results suggest that some PAH promote the secretion of IL-22 and concomitantly inhibit IL-17A production in PBMCs from both healthy and asthmatic donors, independently of the culture cytokine conditions.

### DEP-PAH and B[a]P increase the percentage of IL-17^-^ IL-22^+^ CD4^+^ T cells

Among PBMCs, CD4, CD8, NK, NKT and DCs have been shown to produce IL-22. To evaluate which sub populations were able to express IL-22 in response to PAH stimulation, IL-22 cell origin was determined by flow cytometry following DEP-PAH and B[a]P stimulation. Although cytokine production was different in quantity between AA and NA, there was no significant difference in the percentage of cytokine positive cells between the two groups, therefore the data were mixed. As compared with control stimulated PBMCs, DEP-PAH and B[a]P activation in Th17 conditions induced a significant increase in IL-22-expressing CD4^+^ T cells but not in IL-22-expressing CD8^+^ cells or DCs (Fig 3B). B[a]P activation also significantly decreased the percentage of IL-17-expressing CD4^+^ cells (Fig 3B). IL-22 expression induced by PAH activation in CD4 cells was observed mainly in IL-17 negative CD4^+^ cells (Fig 3B). A representative flow cytometry profile is shown in S2 Fig Similar distribution was obtained in non Th17 conditions (data not shown). No expression of IL-22 and IL-17 was observed in NK cells and monocytes, and NKT cells did not survive in our culture conditions. Thus, among total PBMCs, DEP-PAH- and B[a]P- induced IL-22- positive cells were mainly non Th17 CD4^+^ T cells.

### B[a]P and DEP-PAH inhibit *RORC*, *RORA* and *NOTCH* whereas they increase AhR target gene *CYP1A1* expression

**Fig 4:**
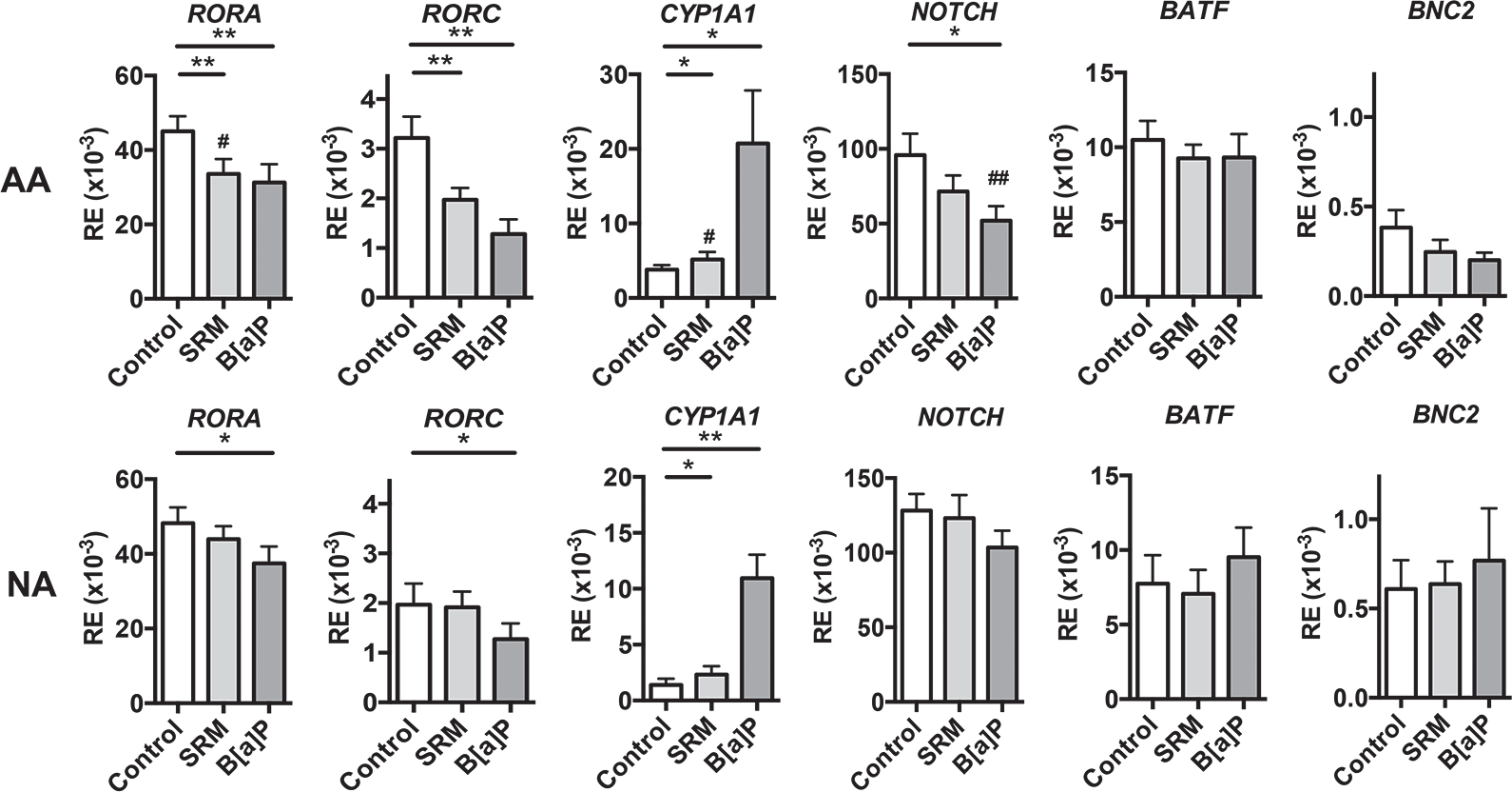
Transcript levels of genes involved in Th17/Th22 polarization. Activated PBMCs from nonallergic (NA) subjects (n = 8) and allergic asthmatic (AA) patients (n = 11) were incubated with or without PAH for 72hr, and gene mRNA level was assessed by Q-RT-PCR. Results are expressed as mean relative expression (RE) of 2^(-ΔCt) ± SEM, where the ΔCt value of the sample was determined by subtracting the Ct value of the target gene from the Ct value of the rs9 house keeping gene. *P<.05,**P<.01, ^#^ *P*<.05 and ^##^ *P* <.01 AA versus NA subjects.

To understand the mechanisms underlying the modified cytokine profile in response to PAH, a number of Th17/Th22-related transcriptions factors were evaluated. Besides the key lineage transcription factors RORα and RORc for Th17 cells and AhR for Th17/Th22 cells, BATF and Notch have been associated to Th17 [33,34] and BNC2 and Notch to Th22 differentiation [15,35]. In agreement with the cytokine protein data, SRM stimulation inhibited *RORA* and *RORC* only in AA patients, and induced AhR target gene *CYP1A1* expression in both groups (Fig 4). The differential effect observed for B[a]P on IL-17 and IL-22 production was reflected at the transcriptional level, with inhibition of *RORA* and *RORC* in both groups and of *NOTCH* only in AA patients, and increases in AhR target gene *CYP1A1* in both groups (Fig 4). In contrast, *BATF* and *BNC2* were not modified. Collectively, these results show that PAH-induced down regulation of IL-17A paralleled transcriptional inhibition of *RORA* and *RORC*, as well as *NOTCH* in AA patients, whereas upregulation of IL-22 resembled activation of AhR.

### IL-22 induction by DEP-PAH is dependent upon AhR

**Fig 5:**
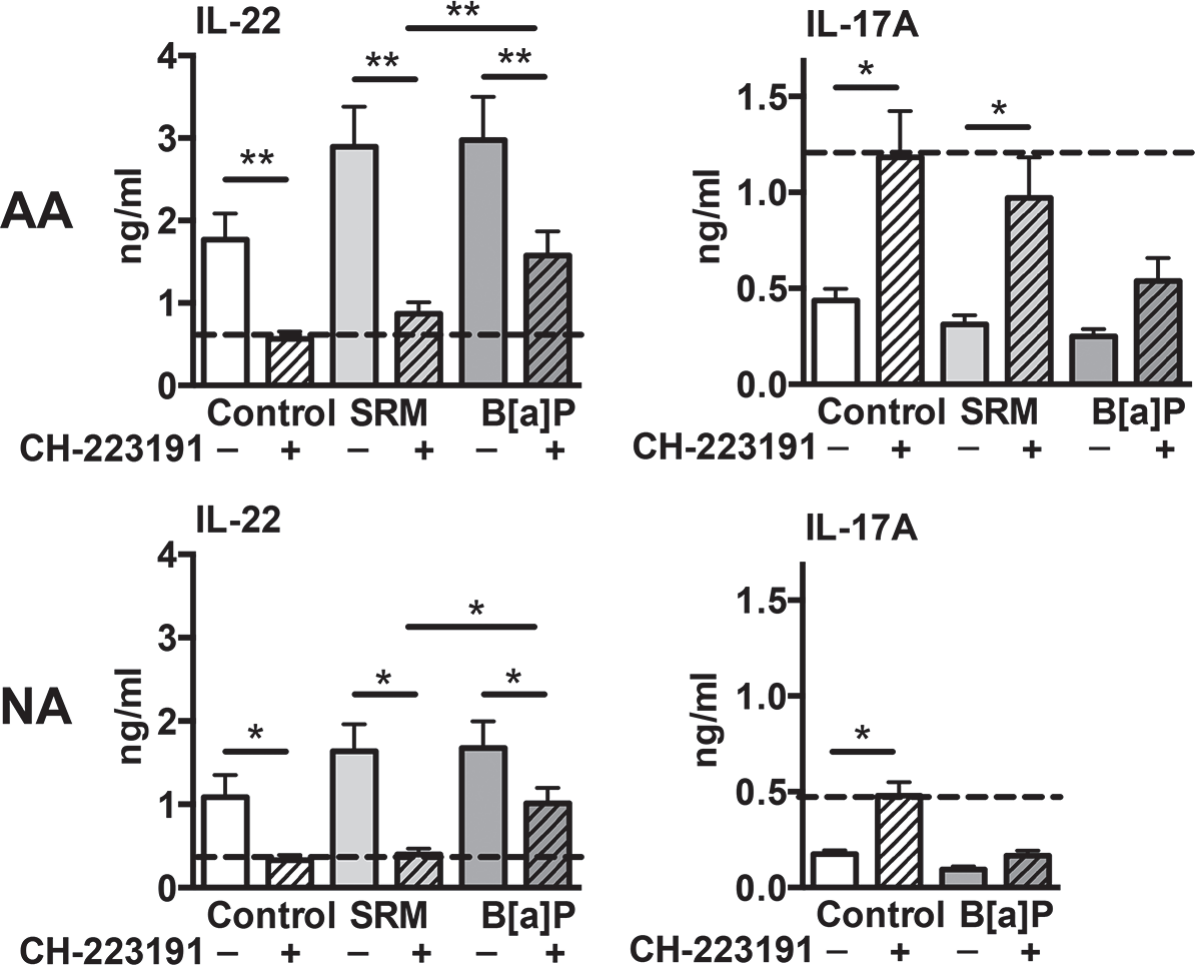
Effects of AhR antagonist on IL-22 and IL-17A secretion by peripheral blood mononuclear cells from Allergic asthmatics (AA) and nonallergic subjects (NA). Activated peripheral blood mononuclear cells from NA subjects (n = 10) and AA patients (n = 12) were incubated or not with PAH, in the presence or not of AhR antagonist CH-223191 for 72hr, and cytokine production was assessed by ELISA in the supernatants. Results are expressed as mean ± SEM. *P<.05, **P<.01. The dotted line is set on the level of the antagonist-treated control cells.

To determine the contribution of AhR to IL-17 and IL-22 regulation by PAH, we used the specific AhR antagonist CH-223191 [29]. Activated control cells treated with CH-223191 exhibited decreased production of IL-22 (Fig 5), consistent with previous observations that endogenous AhR ligands are induced by anti-CD3/CD28 stimulation and present in culture medium [19,26]. In both groups, IL-22 induced production in SRM-stimulated conditions was almost completely inhibited by the AhR antagonist, reaching the same level as antagonist-treated control cells (dotted line) (Fig 5) showing that both endogenous and exogenous AhR-induced IL-22 production was AhR dependent. In B[a]P-stimulated conditions, the enhancing effect on IL-22 secretion was only half reversed by the AhR antagonist at levels corresponding to the inhibition of the endogenous AhR-induced IL-22 alone. In activated control cells, AhR antagonist induced increased level of IL-17A in both groups (Fig 5), but no significant modifications of IL-17F, CCL18 (S3 Fig) and CCL20 secretion (data not shown). As SRM stimulation induced IL-17 modifications only in AA patients (Fig 2), the antagonist was tested in this condition only in these patients. Upon SRM stimulation, IL-17A production in AA was significantly restored by the antagonist but not to the level of the antagonist-treated control cells (dotted line) (Fig 5). In B[a]P-stimulated conditions, the inhibitory effect on IL-17A production was not reversed by the AhR antagonist in both groups (Fig 5). The effect of B[a]P on CCL18 production in both groups and on IL-17F in AA patients was not dependent on AhR (S3 Fig). Altogether, these results suggest that AhR is a major pathway in DEP-PAH- induced IL-22 production, whereas other pathways might contribute to B[a]P-induced IL-22/IL-17A imbalance.

### PI3K, JNK and ERK participate in the enhancing effect of B[a]P on IL-22 production

**Fig 6:**
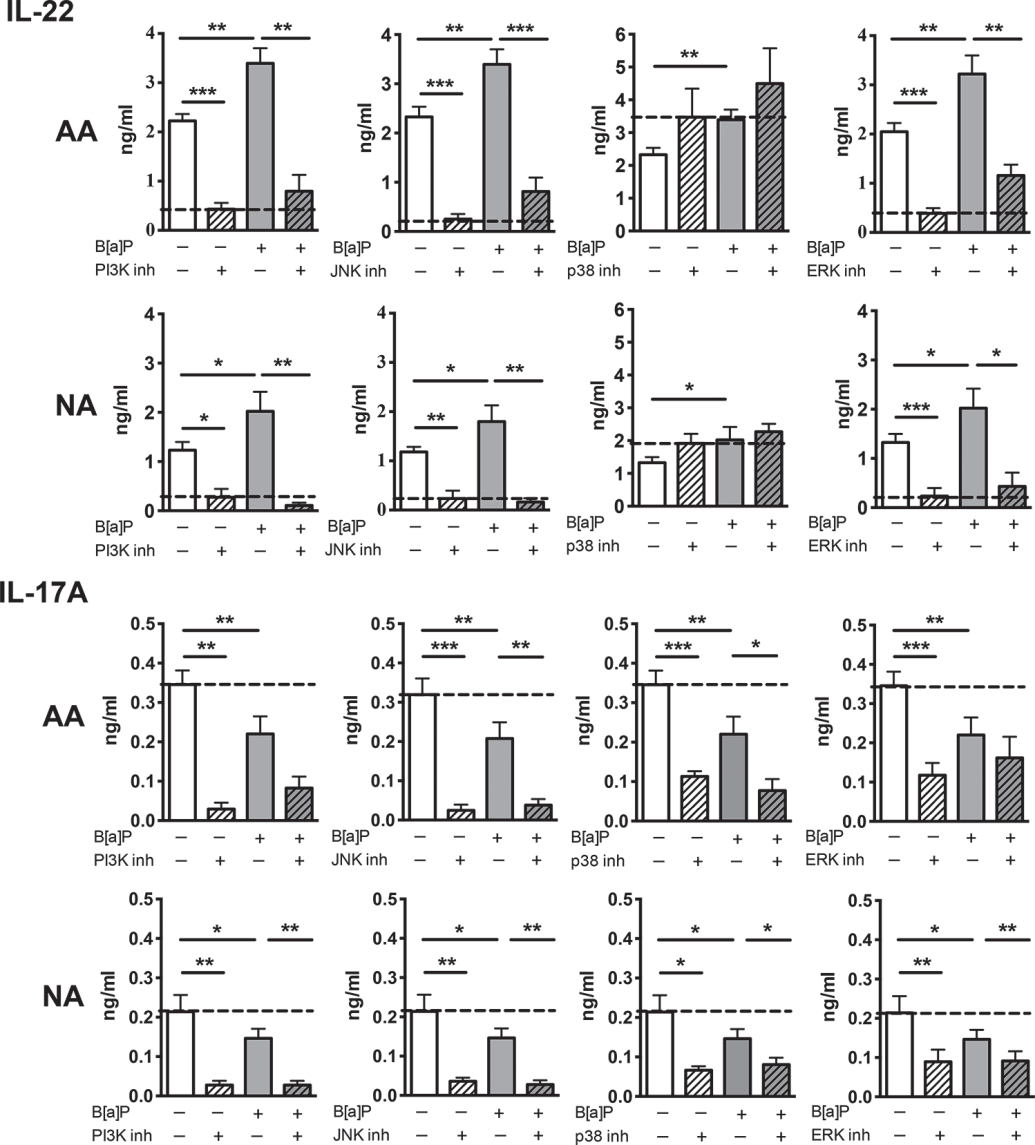
Effects of kinase inhibitors on IL-22 and IL-17A secretion. Activated peripheral blood mononuclear cells from nonallergic (NA) subjects (n = 8) and allergic asthmatic (AA) patients (n = 8) were incubated or not with PAH and with or without inhibitors of kinases for 72hr, and cytokine production was evaluated by ELISA in the supernatants. Results are expressed as mean ± SEM. *P<.05, **P<.01 ***P<.001. The dotted line is set on the level of inhibitor-treated control cells for IL-22 and of control cells for IL-17 and indicates the level to achieve for complete dependence upon the pathway evaluated.

B[a]P has been shown to have effects independent of the AhR pathway through the activation of MAPK [36], therefore the involvement of different kinases was investigated in B[a]P-stimulated PBMCs. PI3K and JNK inhibitors totally inhibited IL-22 induction in B[a]P-stimulated cells in NA subjects, and less importantly in AA patients (Fig 6). ERK inhibitor also displayed a potent inhibitory effect on B[a]P induced IL-22, again more important in NA than in AA subjects. In contrast, p38 inhibitor did not significantly modify IL-22 production in either group (Fig 6). For IL-17A production, the inhibitory effect induced by B[a]P was not restored to the level of control cells but was reduced with PI3K, JNK p38 and ERK inhibitors showing that these kinases are in fact inducers of IL-17A, and are not involved in IL-17A inhibition by BaP (Fig 6). Overall, these data suggest that PI3K JUNK and ERK are involved in IL-22 induction by B[a]P, whereas IL-22 induction by DEP-PAH is mainly mediated by AhR.

## Discussion

The aim of this study was to evaluate the involvement of PAH in IL-22 and IL-17 production in asthmatic patients, as recent studies point out a participation of these cytokines in some endpoints of asthma. Without PAH stimulation, anti-CD3/CD28-activated T cells from AA patients exhibited higher expression of the master Th17 transcription factors *RORC* and of the AhR target gene *CYP1A1* than NA subjects, which were associated with increased levels of IL-17A, IL-17F and IL-22 cytokines, suggesting that AA patients might display a higher sensitivity to the induction of the Th17/Th22 profile than NA subjects after polyclonal activation. However, there was no difference in the Th2 profile between AA and NA donors after polyclonal stimulation. These data are in agreement with the literature showing that in contrast to antigen-mediated activation, polyclonally stimulated PBMCs exhibit similar IL-13 and IL-4 production in allergic and control subjects [37,38].

DEP-PAH and B[a]P stimulation exacerbated IL-22 secretion but inhibited the production of IL-17A. These data are in contrast to mouse studies where the AhR ligands TCDD and FICZ induce both cytokines [6–8], indicating possible species-specific effects of AhR agonists. Accordingly, differences in affinities of the mouse and human AhR have been observed for their ligands [39]. In human studies, mainly differential regulation of IL-17A and IL-22 has been previously observed [7,19,20]. In all these human studies, Th17 type cytokines were used in the culture conditions, however In the present study, the same differential effect on IL-17 and IL-22 was observed whatever the presence or absence of Th17 differentiating cytokines. This differential regulation was observed in both asthmatic and healthy subjects suggesting that they were not related to the subject status. Another difference between all these studies, including ours, is the use of different AhR agonists, which may explain the different outcomes, as the mechanisms mediating the effects of AhR ligands still remain elusive and controversial [40]. It is of note that although the chosen PAH have been shown to enhance allergen-induced experimental asthma [27,28,41], only DEP-PAH and BaP modulated IL-17A and IL-22 production, showing that not all AhR ligands behave similarly. Studies on human Th17 differentiation have revealed that absence of TGF-β1 inhibits IL-17 while promoting IL-22 production [24]. However, in our conditions, PAH stimulation did not modify TGF-β1 secretion, ruling out such a mechanism. Similarly, although some AhR ligands such as TCDD have been shown to favour IL-10 producing regulatory T cells in humans [22], PAH stimulation did not modify IL10 production and did not induce CCL18, a chemokine with IL-10 dependent regulatory effect [25,31,32]. IL-22 originated mainly from CD4 T cells, which did not express IL-17, suggesting that Th17 cells were not the origin of PAH-induced IL-22 intra cellular expression. It is of interest that multiparameter hierarchical cluster analysis has highlighted the fact that in humans T cells, IL-22 producing T cells should be considered independently from the Th17 and Th1 subsets [42]. These IL-22 producing T cells may be Th22 cells, as only few of them expressed intra cellular IL-4 (around 2%) or IFN-γ (around 5%). Although AhR is strongly expressed by DCs [43], these cells did not contribute to PAH-induced IL-22 production in PBMCs. We then evaluated the transcriptional events occurring after PAH activation. In mice, the transcription factors RORγt and RORα, which control the generation of Th17 cells, seem to be the most critical for IL-22 production [10], whereas in human T cells, AhR seems to be the major transcription factor involved in IL-22 production [7]. In our conditions, PAH stimulation led to decreased transcription of *RORA* and *RORC* genes, in agreement with the inhibitory effect observed on IL-17A, and to increased AhR target gene *CYP1A1* expression concomitantly to IL-22 enhancement suggesting that PAH differentially regulate these cytokines at the transcriptional level in humans. Among the other genes evaluated, *NOTCH* expression appeared associated with the inhibition of IL-17A, but only in AA patients, suggesting an involvement of this pathway specifically in these patients. In favour of this hypothesis, polymorphisms of *NOTCH* have been associated with asthma [44]. The role of AhR was evaluated using a specific antagonist. It inhibited IL-22 production, but it also increased endogenous AhR ligand-induced IL-17A production, again suggesting an antagonistic regulation of these two cytokines by AhR, at least in PBMCs and in response to endogenous ligands. Concerning exogenous ligands, DEP-PAH-induced IL-22 production was totally dependent upon AhR, as the antagonist compound abolished the entirety of the endogenous and exogenous AhR- induced IL-22 production, whereas B[a]P-induced IL-22 secretion involved mainly kinases. It is of note that the kinase inhibitors did not differentially regulate IL-22 and IL-17 and were not involved in IL17 inhibition. Thus, it is not clear what mechanisms drive the full inhibition of IL17 production by PAH, and this will need further investigation.

Animal models of asthma have shown that IL-17A and IL-22 can be deleterious or protective. IL-17A can mediate steroid resistant inflammation and airway hyperresponsiveness [45] whereas it can also negatively regulate established asthma [46]. IL-22 has been shown to contribute to allergic asthma during the sensitization phase [17] and to promote lung immunopathology during fungal allergy [47], but it can also inhibit antigen-induced eosinophil airway inflammation through inhibition of IL-25 [48], of IL-10 [49], or during the challenge phase [17]. Interestingly, this ambivalence may relate to dose dependent effects, as IL-17A at low and high doses exhibit opposite effects on Th2 airway inflammation with respectively an increase and a decrease in eosinophil infiltration [50]. Similarly, in a model of epithelial tumorigenesis, IL-22 induction at baseline is protective, whereas sustained high levels of IL-22 cause prolonged epithelial proliferation promoting intestinal tumours [51]. In our study, induction of IL-22 by PAH was similarly observed in healthy and asthmatic donors but the final level was higher in asthmatic subjects. In humans, increase in IL-22 has been associated with more severe asthma [18], and IL-22 enhances human airway smooth muscle cell hyperplasia [11,12], epithelial-mesenchymal transition in asthmatic bronchial epithelial cells [52], as well as cutaneous remodeling [15]. Taken together, these studies suggest that limited or enhanced production of IL-22 might respectively limit cell infiltration and promote tissue repair, including potentially airway remodeling, although this requires further evaluation. Altogether, these data suggest that some PAH might contribute to increased IL-22 production in both healthy and asthmatic patients, through mechanisms involving both AhR- dependent and independent pathways.

## Supporting Information

S1 Fig Cytokine profile of PAH-stimulated PBMCs. IL-17F, TGF-β1 and CCL18 secretion by activated PBMCs from nonallergic (NA) subjects (n = 10) and allergic asthmatic (AA) patients (n = 12) incubated or not with PAH. Results are expressed as mean ± SEM. *P<.05 and ***P<.001 versus control.
(TIF)

S2 Fig Representative flow cytometry experiment gated on the CD4 subset. (TIF)

S3 Fig Effects of AhR antagonist on IL-17F and CCL18 secretion by PBMCs. Activated PBMCs from nonallergic (NA) subjects (n = 10) and allergic asthmatic (AA) patients (n = 12) were incubated or not with PAH, in the presence or not of AhR antagonist CH-223191. The dotted line is set on the level of the antagonist-treated control cells. Results are expressed as mean ± SEM.
(TIF)

S1 Table Patients’ characteristics. (DOC)

S2 Table Primer oligonucleotide sequences. (DOC)
