# Supplementary figures and images for: Polycyclic Aromatic Hydrocarbons Reciprocally Regulate IL-22 and IL-17 Cytokines in Peripheral Blood Mononuclear Cells from Both Healthy and Asthmatic Subjects
Source: PLoS One. 2015 Apr 10;10(4):e0122372. doi: 10.1371/journal.pone.0122372 (PMC4393221; doi:10.1371/journal.pone.0122372)

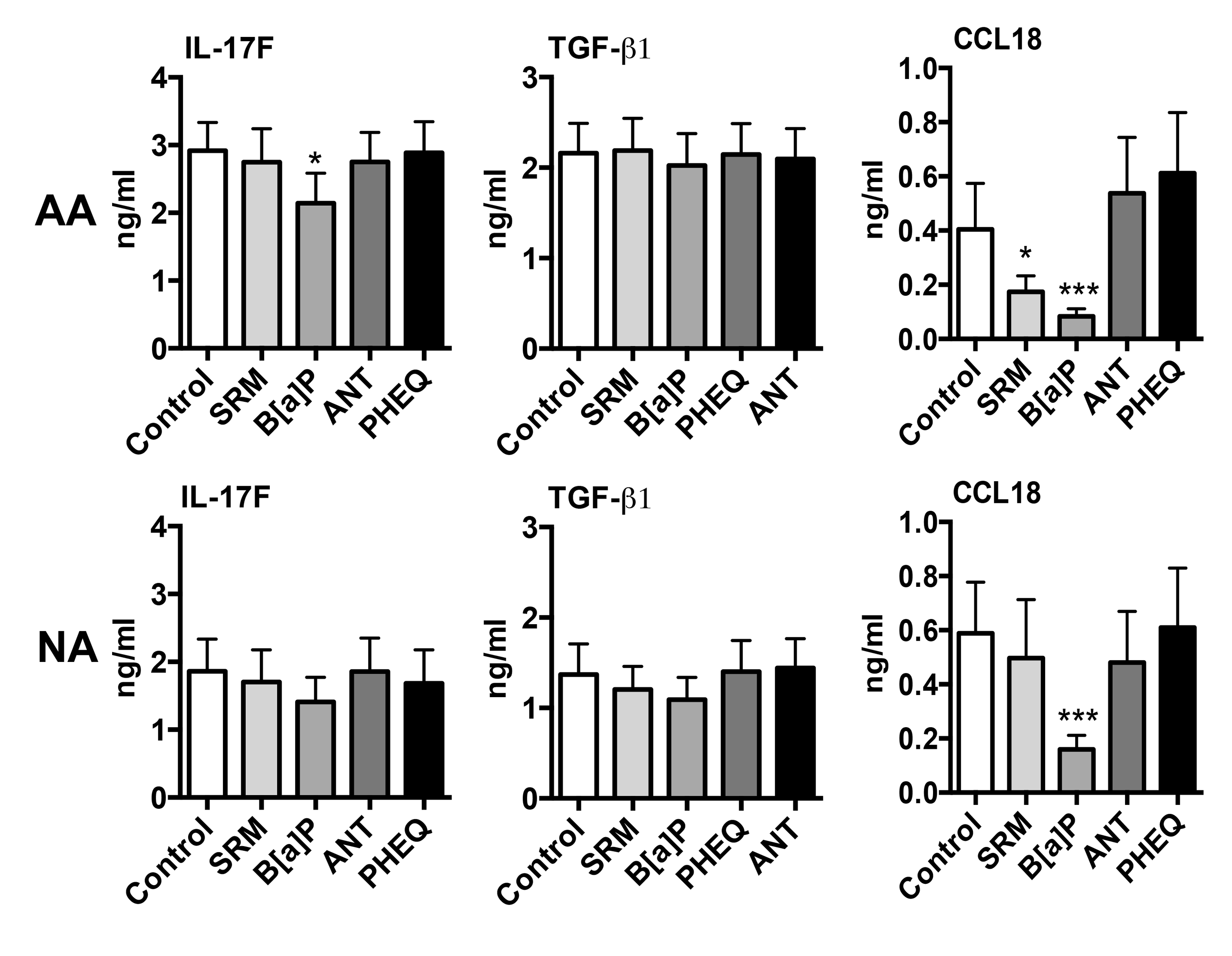

Supplement: S1 Fig — IL-17F, TGF-β1 and CCL18 secretion by activated PBMCs from nonallergic (NA) subjects (n = 10) and allergic asthmatic (AA) patients (n = 12) incubated or not with PAH. Results are expressed as mean ± SEM. *P<.05 and ***P<.001 versus control. (TIF) [file pone.0122372.s001.tif]

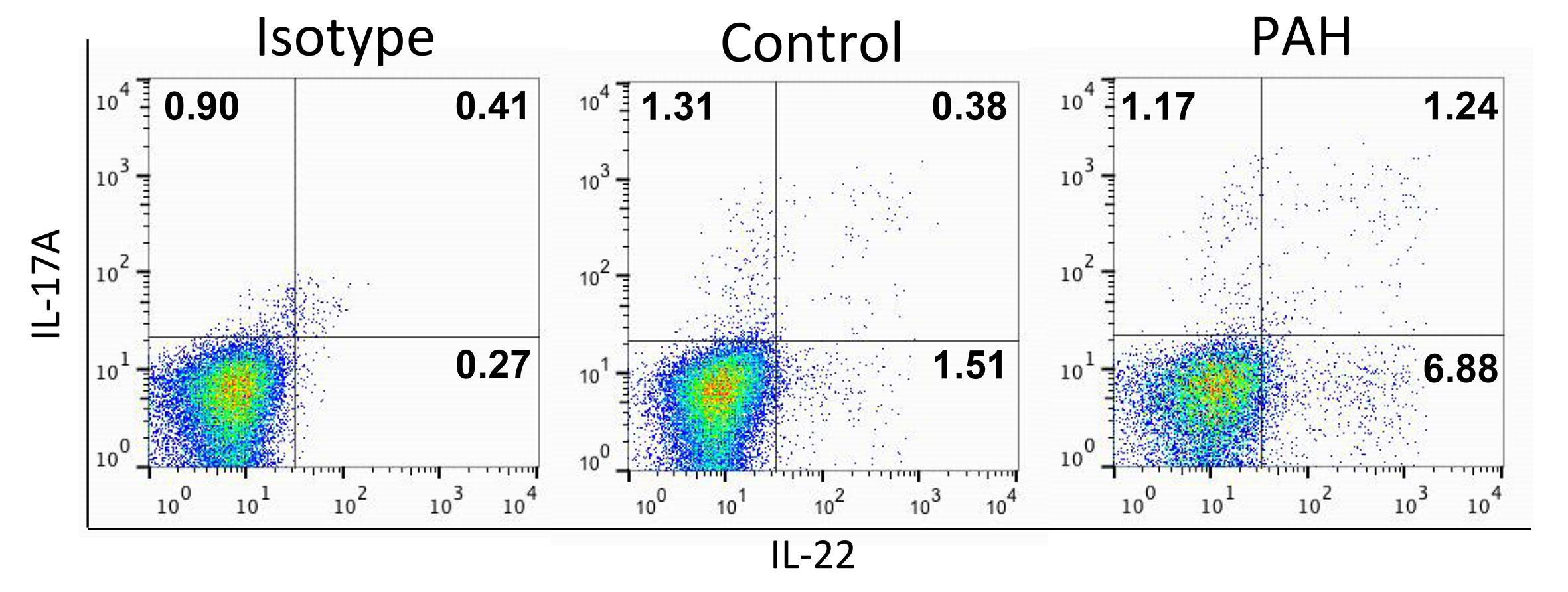

Supplement: S2 Fig — (TIF) [file pone.0122372.s002.tif]

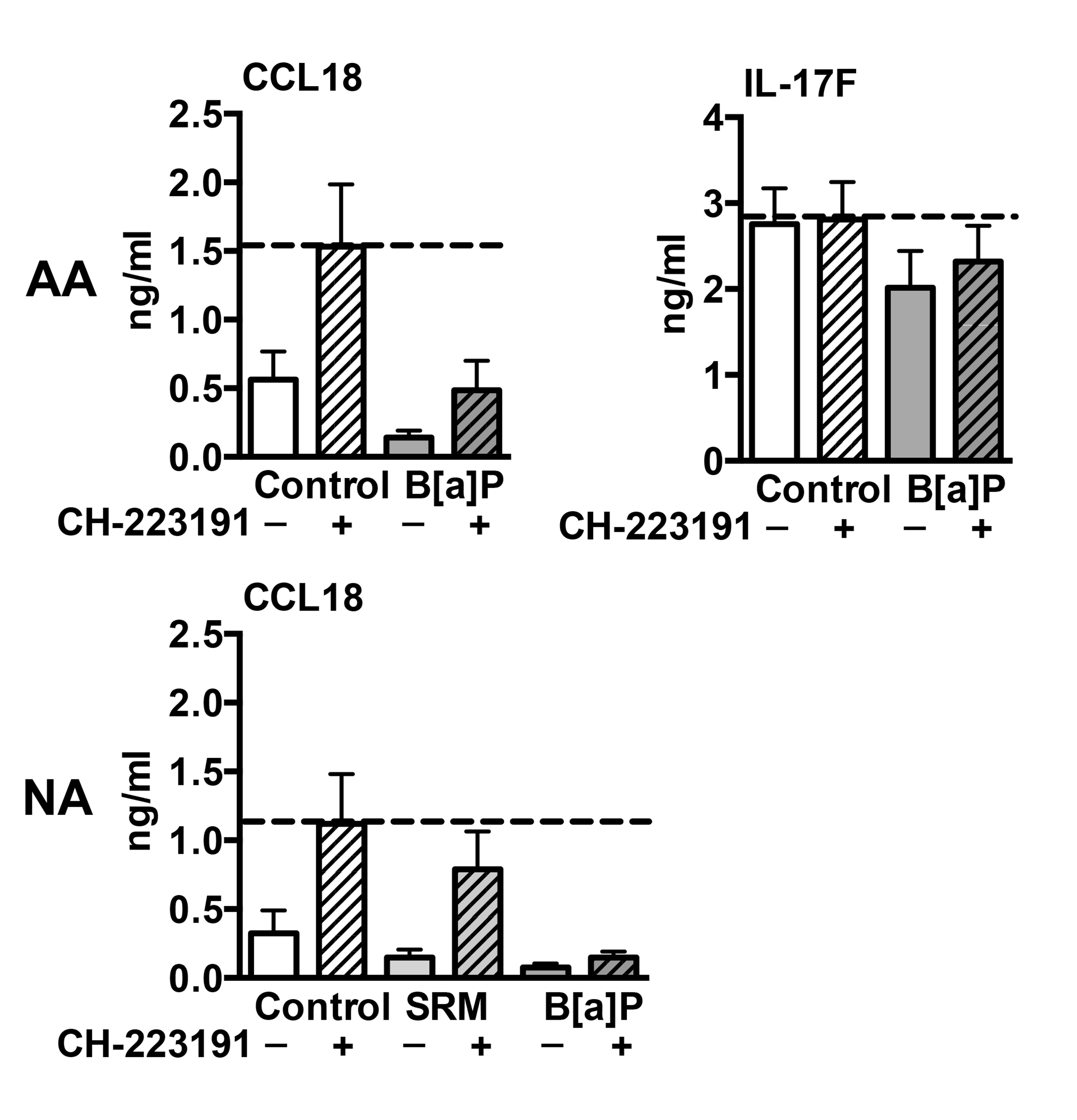

Supplement: S3 Fig — Activated PBMCs from nonallergic (NA) subjects (n = 10) and allergic asthmatic (AA) patients (n = 12) were incubated or not with PAH, in the presence or not of AhR antagonist CH-223191. The dotted line is set on the level of the antagonist-treated control cells. Results are expressed as mean ± SEM. (TIF) [file pone.0122372.s003.tif]
