# Supplementary material for: Polycyclic Aromatic Hydrocarbons Reciprocally Regulate IL-22 and IL-17 Cytokines in Peripheral Blood Mononuclear Cells from Both Healthy and Asthmatic Subjects
Source: PLoS One. 2015 Apr 10;10(4):e0122372. doi: 10.1371/journal.pone.0122372 (PMC4393221; doi:10.1371/journal.pone.0122372)
Supplement: S1 Table — (DOC) [file pone.0122372.s004.doc]

**Table S1 : Patients’ characteristics**

|  | Healthy control  subjects | Asthmatic  patients | P values |
| --- | --- | --- | --- |
| Sex (F/M) | 8/8 | 8/8 |  |
| Age (y) | 40.3 ± 3.59 | 34.8 ± 2 .64 | 0.20 |
| Duration of asthma (y) | NA | 13.4 ± 2.6 |  |
| FEV1 (% predicted) | 97.1 ± 1.84 | 89.6 ± 2.69 | 0.03 |
| FEV1/FVC ratio (%) | 79.5 ± 0.85 | 75.36 ± 1.46 | 0.003 |
| Atopy | 0/16 | 16/16 |  |
| Treatment | None | Beta2 agonists as needed |  |
| Smoking  Socio economic status  (low/Middle/high class)  BMI above 25 | 0/14  2/7/7  0/16 | 0/16  3/8/5  0/16 |  |

Data are presented as n or mean ± SEM, unless otherwise stated. Y: year; FEV1: forced expiratory volume in 1 second; FVC: forced vital capacity; NS: not significant. Low class: <1500 €/month; Middle class: 1501-3000 €/month; High class: >3000 €/month. BMI: body mass index (kg/m^2^).
