# Supplementary material for: Polycyclic Aromatic Hydrocarbons Reciprocally Regulate IL-22 and IL-17 Cytokines in Peripheral Blood Mononuclear Cells from Both Healthy and Asthmatic Subjects
Source: PLoS One. 2015 Apr 10;10(4):e0122372. doi: 10.1371/journal.pone.0122372 (PMC4393221; doi:10.1371/journal.pone.0122372)
Supplement: S2 Table — (DOC) [file pone.0122372.s005.doc]

Table S2. Primer oligonucleotide sequences

|  | |
| --- | --- |
| **mRNA** | **Oligonucleotide sequences** |
| *BATF* | Forward: 5' - ACGCAGGGGTCAGAGGTGGCTACA -3’  Reverse: 5'- GGCTCTTCTGGGCGGCAATACGA -3' |
| *BNC2* | Forward: 5' - GACTCGTCGCAGCCCTCCCTTAG -3'  Reverse: 5'- ACTGTCACTCGCCCCGTCAATGTC -3' |
| *CYP1A1* | Forward: 5’- GGTCAAGGAGCACTACAAAACC -3’  Reverse: 5’- TGGACATTGGCGTTCTCAT -3’ |
| *NOTCH2* | Forward: 5' - CTGGCTTTGCTGGGGAGCGTT -3'  Reverse: 5'- TCCCGGGGGACAACGGCAAA -3' |
| *RORA* | Forward: 5' - ATGCCTTGCCGTAGGGATGT -3'  Reverse: 5'- CCGTTGGCCGAGATGTTGTA -3' |
| *RORC* | Forward: 5' - GCATGTCCCGAGATGCTGTC -3'  Reverse: 5'- CTGGGAGCCCCAAGGTGTAG -3' |
| *RS9* | Forward: 5' - AAGGCCGCCCGGGAACTGCTGAC -3'  Reverse: 5’- ACCACCTGCTTGCGGACCCTGATA -3’ |
